# Intrathoracic ectopic kidney with pulmonary sequestration: clinical and surgical insights: a case report

**DOI:** 10.1093/jscr/rjae757

**Published:** 2024-12-03

**Authors:** Isam Ahmed Abdeljaleel Taha, Mohamed Y Ibrahim, Mohamed Helali, Sami Mohamed Elamin Taha, Baha Aldeen Alshareif, Hala Altayeib, Razan Idriss, Esra S Abdalgadir

**Affiliations:** Department of Pediatric Surgery, Pediatric Surgery Center, National Ribat University Hospital, Khartoum, Sudan; Pediatric Surgery Center, National Ribat University Hospital, Khartoum, Sudan; Pediatric Surgery Center, National Ribat University Hospital, Khartoum, Sudan; Department of Pediatric Surgery, Sidra Medicine-Qatar, Doha, Qatar; Department of Surgery, Faculty of Medicine, Alzaiem Alazhari University, Khartoum, Sudan; Pediatric Surgery Center, National Ribat University Hospital, Khartoum, Sudan; Pediatric Surgery Center, National Ribat University Hospital, Khartoum, Sudan; Pediatric Surgery Center, National Ribat University Hospital, Khartoum, Sudan

**Keywords:** ectopic kidney, intrathoracic ectopic kidney, sequestrated lung lobe

## Abstract

Intrathoracic ectopic kidney is an extremely rare congenital defect that is frequently identified by accident because it is asymptomatic. Even more unusual is its link to pulmonary sequestration alone. This case report describes the clinical presentation of a 7-month-old child with a history of recurrent respiratory distress and chest infections since birth, who had shortness of breath, failure to thrive, and delayed developmental milestones. The clinical examination and imaging revealed an ectopic right kidney in the thoracic cavity, as well as a probable sequestered lung lobe. Thoracotomy revealed a healthy right lung with a collapsed lower lobe connected to the ectopic kidney. The right lower third lobe was released to be in its normal appearance and the sequestration vessels were ligated. The patient recovered smoothly after surgery, with the exception of a brief episode of pneumonia that was treated. The patients follow up confirmed complete recovery without complications.

## Introduction

Intrathoracic ectopic kidney is a relatively rare type of ectopic kidney, with a documented occurrence of roughly 5 per million live births and an incidence of less than 5% of all ectopic kidneys [1, 2]. In such cases patients usually present with no symptoms and discovered incidentally as an opacity in chest radiography [3, 4]. The majority of instances reported in the literature include children aged 3–14. Renal ectopia can occur in a variety of places, including the pelvis, iliac fossa, abdomen, and thorax. However, because the intrathoracic ectopic kidney appears as opacity or lobar consolidation on chest X-rays, it may be mistakenly considered to be pneumonia [5, 6]. Surgical treatment is needed for patients experiencing symptomatic or severe breathing problems and other serious congenital abnormalities [7, 8].

Pulmonary sequestration on the other hand is also a congenital malformation, which is nonfunctioning lung tissue supplied with systemic blood. The two types of pulmonary sequestration that can be differentiated based on where they are found are extralobar (pulmonary parenchyma separate from the lungs) and intralobar (situated in the lung lobe). The symptoms that may be present may include cough, sputum, fever, and hemoptysis. Diagnosis is usually made preoperatively. Chest radiography and CT are most often applied to reveal the presence of features like solid-cystic masses and the variant arterial blood supply. Thoracotomy and lobectomy are standard for addressing different pathological processes, and after the interventions, patients encounter minimal postoperative comorbidity [9, 10].

The verity of an intrathoracic kidney and pulmonary sequestration alone is an extraordinary unusual syndrome that has never been stated in Sudan. We present an exceptional instance of a right thoracic kidney coexisting with a sequestered lung lobe that was not accompanied by a diaphragmatic hernia.

## Case report

**Figure 1:**
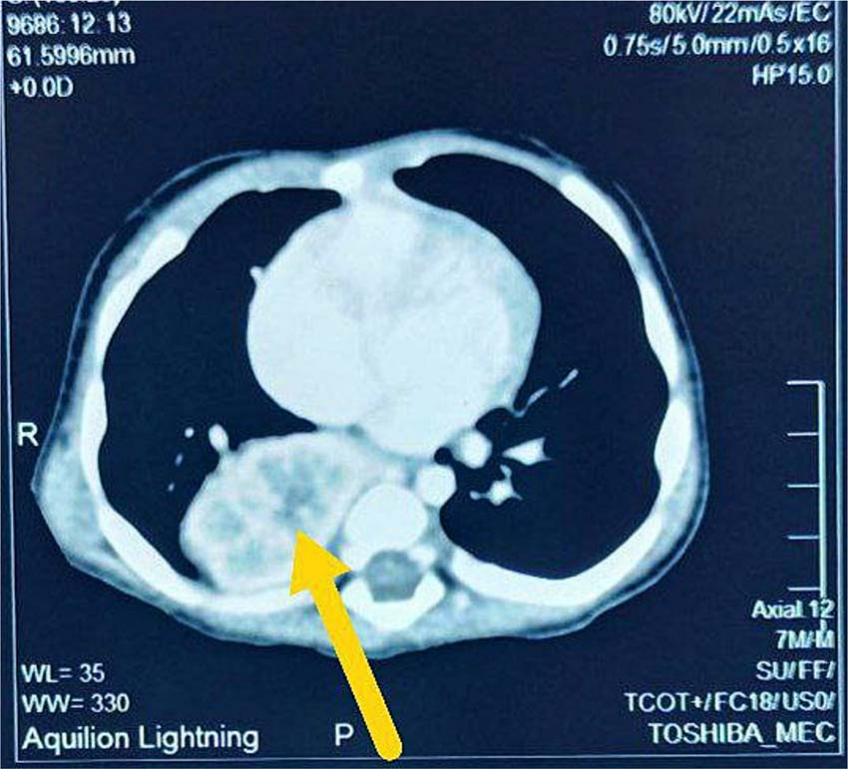
Axial view CT scan shows right intrathoracic kidney (arrow).

**Figure 2:**
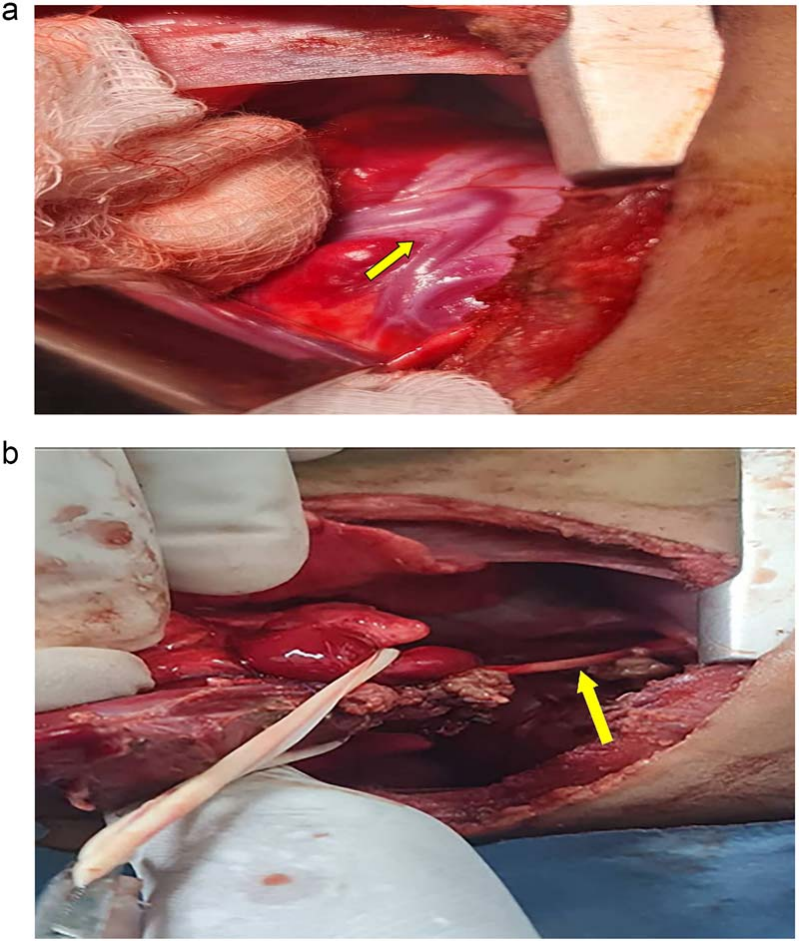
(a) Shows the sequestration feeding artery (arrow). (b) Shows the dissected feeding artery traversing the right hemidiaphragm originating from the abdominal aorta.

**Figure 3:**
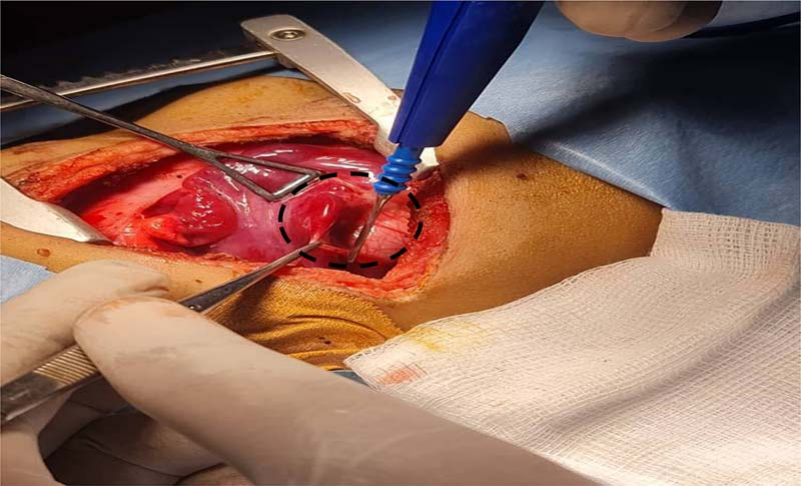
Shows the sequestrated lung lobe dissected off the fascia of gerota (shown by interrupted line).

**Figure 4:**
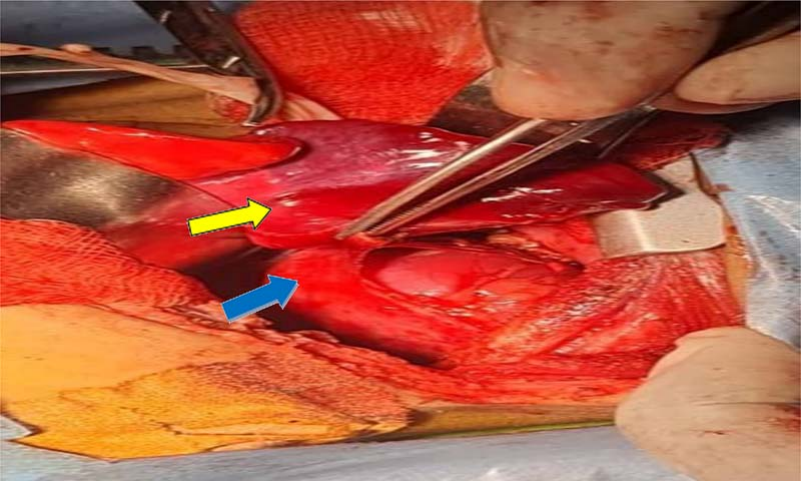
Shows dissection of the sequestration (yellow arrow) and ectopic kidney after separation from fascia of gerota (can see the renal capsule) (blue arrow).

**Figure 5:**
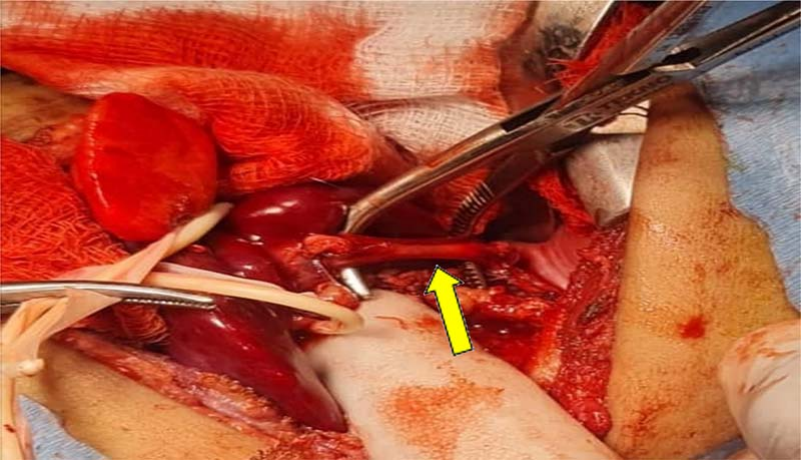
Shows the dissected feeding artery.

**Figure 6:**
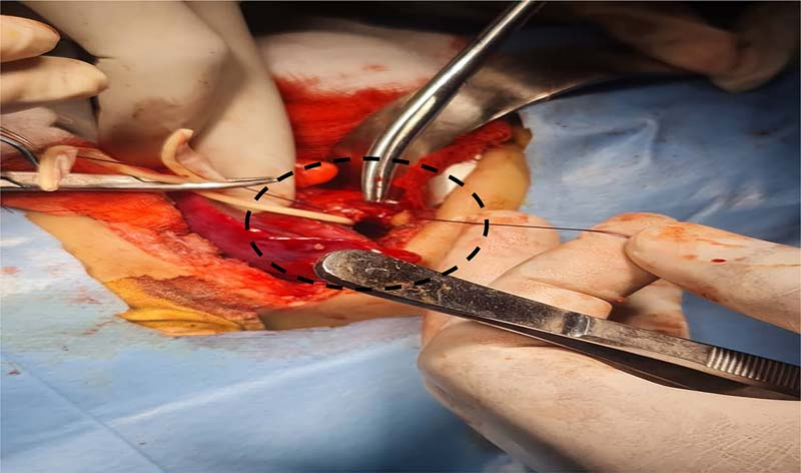
Illustrates ligation of feeding artery.

**Figure 7:**
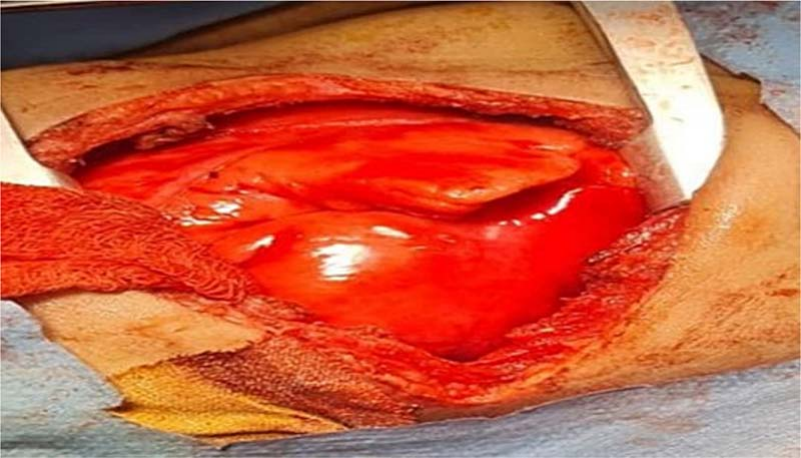
Shows inflation of the previously hyperemic sequestrated lung lobe after ligation of the feeding vessel.

A 7-month-old infant with recurrent hospital admissions for shortness of breath during breastfeeding and recurrent chest infections since birth. He is the fourth child of a mother who had a normal pregnancy and received good antenatal care. Despite a full-term delivery with normal amniotic fluid levels and immediate postnatal crying and passage of meconium, the infant displayed delayed developmental milestones, failure to thrive, and breast milk refusal. Physical examination revealed signs of respiratory distress, including intercostal retractions and diminished right-sided breath sounds. A computed tomography (CT) scan of the chest revealed the right kidney located in the right thoracic cavity, consistent with an intrathoracic kidney (Fig. 1). The presence of a natural opening through which the ureter traversed the diaphragm supported this diagnosis. A mass lateral to the kidney was suspected to be a sequestrated lung lobe. Laboratory investigations returned within normal limits. Differential diagnoses considered congenital diaphragmatic hernia and diaphragmatic eventration. Subsequent thoracotomy revealed an intact right lung, with the ectopic kidney’s Gerota’s fascia adhering to the collapsed lower lobe (Figs 2–4). Sequestration vessels were ligated, and the lung lobe was successfully released from the kidney (Figs 5–7). The kidney remained within the thoracic cavity, and the lower lobe was re-expanded. Postoperative management included hemostasis assurance and chest tube insertion, with no immediate complications noted.

## Discussion

Intrathoracic ectopic kidney was first described using retrograde pyelography in 1940 [8]. This condition more prevalent in males than females (2:1), with a left-sided predominance due to the spleen’s reduced barrier action [11, 12]. Intrathoracic ectopic kidneys present with no symptoms causing neither urinary or thoracic complications and determined incidentally as an opacity in chest radiography. Our case is a 7-month-old boy with intrathoracic kidney complicated by sequestrated lung lobe. Adds to the small body of research on this rare combination, highlighting the challenges in diagnosis and treatment.

### Clinical presentation and diagnosis

The patient’s clinical history of repeated respiratory distress and chest infections since birth, along with failure to thrive, indicated a serious underlying condition. The initial differential diagnoses included typical pediatric respiratory illnesses; however, the persistence of symptoms despite treatment necessitated additional inquiry. Imaging examinations, particularly a CT scan, revealed the right kidney in the thoracic cavity, next to a mass thought to be a sequestrated lung lobe. The diagnosis of an intrathoracic kidney was supported by the existence of natural holes through which the ureter passed into the thoracic cavity.

The abnormal ascent of the kidney during fetal development is often responsible for intrathoracic kidney formation [13]. Unlike congenital diaphragmatic hernia, which occurs when abdominal contents herniate into the thoracic cavity through a diaphragmatic defect, the intrathoracic kidney typically has a structurally intact diaphragm, but with a natural or unusual opening allowing the ureter to pass through [14]. In this case, the CT results and surgical findings confirmed the diagnosis of an intrathoracic kidney without diaphragmatic hernia, highlighting the rarity of this presentation.

### Surgical intervention and outcomes

The surgical approach through thoracotomy showed a healthy right lung with a deflated lower lobe attached to the kidney, indicating pulmonary sequestration. Pulmonary sequestration, particularly intralobar sequestration, is a congenital abnormality characterized by non-functioning lung tissue and an abnormal arterial supply from the systemic circulation. This condition present as recurring infections and respiratory discomfort, which are identical to the symptoms detected in this case. The effective removal of the sequestrated lobe from the kidney and ligation of the abnormal arteries restored the normal function of the lungs, with the lower lobe inflating effectively after the treatment. The decision was made to leave the kidney in the thoracic cavity because the patient had steady renal function and no obstructive symptoms. This choice is in accordance with current management recommendations, which advise conservative treatment for intrathoracic kidneys that are asymptomatic unless problems develop. Everything went well during the postoperative recovery period except for a brief episode of pneumonia that was treated well with antibiotics. Subsequent monthly follow-up visits with a pulmonologist verified complete recovery with no new complications, highlighting the effectiveness of the surgical procedure.

### Implications for clinical practice

This case highlights the need of including rare congenital defects in the differential diagnosis of chronic respiratory symptoms in pediatric patients. Previous exact diagnosis with advanced imaging intervention is required for remedy. Furthermore, the good surgical outcome in this patient highlights the importance of developing individualized treatment strategies based on each patient’s specific anatomical and functional needs.

## Conclusions

Intrathoracic ectopic kidney, especially when accompanied by pulmonary sequestration, presents a distinct therapeutic difficulty. This case highlights the importance of increased clinical awareness and a multidisciplinary approach to ensuring the optimal possible outcomes. The continuous documentation and reporting of such rare incidents is essential to increase awareness, improving understanding and management of these complex congenital abnormalities.
